# Fecal carriage of multiresistant bacteria versus infection in ICU wards patients

**DOI:** 10.1186/1753-6561-5-S6-P134

**Published:** 2011-06-29

**Authors:** A Eusébio, A Narciso, L Gonçalves, A Costa, A Godinho, F Fernandes, A Duarte

**Affiliations:** 1 i.Med, Faculdade de Farmàcia, Lisbon, Portugal; 2 SAMS Hospital, Lisbon, Portugal

## Introduction / objectives

Healthcare-associated infections (HAI) constitute major health care problem from their frequency, cost and gravity. Fecal carriage of extended-spectrum beta-lactamase (ESBL)-producing isolates has mainly been detected in HAI, and few studies have evaluated fecal carriage during non-outbreak situations and among patients in the community. This study was undertaken to determine the spectrum of bacterial colonization and predisposing risk factors in patients being admitted to an acute care hospital, Lisboa, with special emphasis on ESBL producing Gram-negative bacteria.

## Methods

To decrease HAI some active preventive measures were taken since November to February 2010. Nasal, oral and rectal swab samples were collected and processed for isolation of ESBL on chromID ESBL (bioMerieux).

## Results

Bacterial colonization of one or more sites on admission was detected in 37 patients included in the study. The most common colonizers were *Escherichia coli* (n=10); *PseudomonasÂ aeruginosa* (n=9); *Klebsiella pneumoniae* (n=8) and *Enterobacter cloacae* (n=7), with simultaneous colonization in six patients. Seven patients were colonized and infected (blood, urine and bronchial secretion) with the same specie identified from rectal swab. After one month of admission at ICU two inpatients were infected (catheter and pus) with *P. aeruginosa* and *Stenotrophomonas maltophilia* present at admission in hospital.

## Conclusion

This study alertsÂ medical professionals that should be aware of these isolates, should continue strict hygiene procedures and, additionally, should implement an ESBL screening system, in particular for faecal carriage, in order to prevent possible outbreaks caused by these multi-resistant organisms.

## Disclosure of interest

None declared.

